# Up-regulation of CDK9 kinase activity and Mcl-1 stability contributes to the acquired resistance to cyclin-dependent kinase inhibitors in leukemia

**DOI:** 10.18632/oncotarget.2096

**Published:** 2014-06-12

**Authors:** Yuh-Ying Yeh, Rong Chen, Joshua Hessler, Emilia Mahoney, Amy M. Lehman, Nyla A. Heerema, Michael R. Grever, William Plunkett, John C. Byrd, Amy J. Johnson

**Affiliations:** ^1^ Division of Hematology, Department of Internal Medicine, Comprehensive Cancer Center, The Ohio State University, Columbus, Ohio, USA; ^2^ Department of Experimental Therapeutics, The University of Texas MD Anderson Cancer Center, Houston, Texas, USA; ^3^ Center for Biostatistics, The Ohio State University, Columbus, Ohio, USA; ^4^ Department of Pathology, The Ohio State University, Columbus, Ohio, USA; ^5^ Division of Medicinal Chemistry, College of Pharmacy, The Ohio State University, Columbus, Ohio, USA

**Keywords:** Flavopiridol, Mcl-1, cyclin-dependent kinase 9 (CDK9), cyclin-dependent kinase (CDK) inhibitor, and leukemia

## Abstract

Flavopiridol is a small molecule inhibitor of cyclin-dependent kinases (CDK) known to impair global transcription via inactivation of positive transcription elongation factor b. It has been demonstrated to have significant activity predominantly in chronic lymphocytic leukemia and acute myeloid leukemia in phase I/II clinical trials while other similar CDK inhibitors are vigorously being pursued in pre-clinical and clinical studies. Although flavopiridol is a potent therapeutic agent against blood diseases, some patients still have primary or acquired resistance throughout their clinical course. Considering the limited knowledge of resistance mechanisms of flavopiridol, we investigated the potential mechanisms of resistance to flavopiridol in a cell line system, which gradually acquired resistance to flavopiridol in vitro, and then confirmed the mechanism in patient samples. Herein, we present that this resistant cell line developed resistance through up-regulation of phosphorylation of RNA polymerase II C-terminal domain, activation of CDK9 kinase activity, and prolonged Mcl-1 stability to counter flavopiridol's drug actions. Further analyses suggest MAPK/ERK activation-mediated Mcl-1 stabilization contributes to the resistance and knockdown of *Mcl-1* in part restores sensitivity to flavopiridol-induced cytotoxicity. Altogether, these findings demonstrate that CDK9 is the most relevant target of flavopiridol and provide avenues to improve the therapeutic strategies in blood malignancies.

## INTRODUCTION

Chronic lymphocytic leukemia (CLL) is the most common adult leukemia in the western world and considered incurable. CLL has been characterized as a clonal B-cell disorder attributed to its defective apoptosis and sustained nurture of the tumor microenvironment. It is a progressive disease and has a very heterogeneous clinical course with survival time ranging from months to decades. Patients are treated with the current frontline chemoimmunotherapy that includes purine analogs and rituximab when the disease advances but patients will eventually relapse and further therapy options are limited. [1, 2] The heterogeneity of CLL can be partly accounted for the high frequency of chromosomal abnormalities which have been identified in ~80% CLL cases by fluorescence *in-situ* hybridization (FISH). [3, 4] The common recurrent karyotypic abnormalities include del(17p13.1), del(11q22.3), trisomy 12, del(13q14), and (del6q.21) and have been established in a hierarchical model showing poor survival in patients with del(17p13.1) and del(11q22.3) but advantagous survival for patients with trisomy 12, normal karyotype, and del(13q14) as the sole abnormality. [3, 5]

The lack of effective therapies for CLL has attracted intensive research in the development of new therapeutic approaches for this disease. A notable advancement in this effort has been the introduction of cyclin-dependent kinase (CDK) inhibitors. Flavopiridol is the first in class broad CDK inhibitor effective in decreasing activity of CDK1, CDK2, CDK6, and CDK7 and CDK9 that has entered clinical trials. After considerable schedule optimization, flavopiridol demonstrated clinical activity for CLL and non-Hodgkin lymphoma (NHL). [6-10] Although having a narrow therapeutic window, it has been shown to be effective in relapsed and refractory CLL patients with 40 – 50% response rates in patients with genetically high-risk disease. [9, 11-13] In vitro and in vivo studies by our laboratory and others have shown that flavopiridol mediates potent apoptosis in CLL cells that occurs independent of del(17p13.1) or loss of p53 function. [11, 12, 14] Further studies in CLL and other leukemias suggest that flavopiridol mediates its cytotoxic effects through inhibition of positive transcription elongation factor b (P-TEFb, CDK9/cyclin T) via CDK9 and hence hampering global RNA transcription. Other drug actions of flavopiridol include depletion of anti-apoptotic proteins, such as Bcl-2, Bcl-xL and Mcl-1, down-regulation of X-linked inhibitor of apoptosis protein (XIAP) and survivin, up-regulation of endoplasmic reticulum (ER) stress response and induction of autophagy. [10, 14-17] Long exposure of flavopiridol in lung and ovarian cell lines has shown to induce DNA damage, suggesting that flavopiridol may have other drug actions yet to be identified. [18]

Because of encouraging results in leukemias and NHL, development of flavopiridol continues both as a single agent and in combination with other therapies in clinical trials. Other CDK inhibitors with similar kinase profiles to flavopiridol are also under development. [19] Although flavopiridol shows good efficacy in CLL and other hematologic malignancies, some patients do not respond or eventually relapse. As with all other cancer therapies, CDK inhibitors acquire resistance in clinic but their resistant mechanisms are poorly described and not well understood, in particular in the blood malignancies. The mechanism underlying resistance to flavopiridol has been associated with in vitro overexpression of the ATP-binding cassette half-transporter, *ABCG2*, in human breast cancer cell lines, exhibiting resistance to flavopiridol; however, it is not consistently observed in other spontaneously developed flavopiridol-resistant cell lines, such as ovarian carcinoma cells and colon carcinoma cells. [20-22] Data from our group showed that combination of flavopiridol and inactivation of autophagy either through chloroquine or genetic knockdown, can induce remarkable cellular cytotoxicity in CLL, suggesting autophagy as a potential resistance mechanism for flavopiridol. [15]

To further explore this, we sought to generate the resistant cell line (Flavo-R) to study the resistance mechanism of flavopiridol and exploit this system for better understanding of flavopiridol-targeted pathways. In this study, we demonstrate that cells acquire resistance via the increase of phosphorylation of Ser2 of RNA polymerase II (RNA Pol II) and its upstream kinase activity (CDK9), indicating that flavopiridol primarily exerts its killing effect through inhibition of transcription. Moreover, activation of the mitogen-activated protein kinases MAPK/ERK is shown to contribute to up-regulation of Mcl-1 levels in Flavo-R through Mcl-1 protein stabilization. Genetic knockdown of *Mcl-1* by shRNA in part restores the sensitivity to flavopiridol in these resistant cells. Our investigation also determines that flavopiridol modulates the transcriptional inhibition not only by targeting CDK9 activity but also decreasing its expression. Ineffective reduction of CDK9 protein expression after flavopiridol therapy associates with poor response to flavopiridol in vivo. Altogether, these findings validate CDK9 as a favorable therapeutic target in CLL and up-regulation of the CDK9-associated pathways, including Mcl-1 and RNA transcription machinery contributes to the resistance of flavopiridol.

## RESULTS

### Lymphoid cells acquire non-transporter mediated resistance to flavopiridol

**Figure 1:**
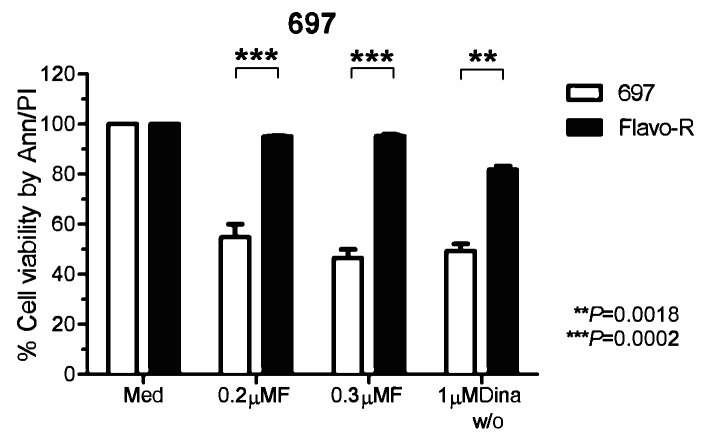
Cells acquired resistance to flavopiridol-induced cell death *in vitro* 697 parental and flavopiridol-resistant (Flavo-R) cells were treated with continuous flavopiridol (0.2μM or 0.3μM), or 1μM dinaciclib with 2-hour exposure and washout (w/o). Cell viability was measured post 24 hours by annexin V-FITC and PI-PE stains, followed by flow cytometry. Flavo-R also develops cross-resistance to dinaciclib with the significant survival advantage over parental cells at 24 hours post to dinaciclib treatment. Due to the similar effects of continuous 0.2μM, 0.3μM flavopiridol, *p*-values represent the average effect for both doses.

Data from our laboratory and others have shown that drug actions of flavopiridol include down-regulation anti-apoptotic proteins, inactivation of P-TEFb (CDK9/cyclin T), and induction of ER stress response and autophagy activity. [10, 14, 15, 23] Hence, to better understand the resistance and drug mechanisms of flavopiridol to improve clinical outcomes, we generated flavopiridol–resistant (Flavo-R) cell lines from initially sensitive pre-B acute lymphoblastic leukemia cell line, 697. Flavo-R is viable with continuous exposure to flavopiridol at concentrations of 500nM. Flavo-R cells are routinely monitored for resistance to flavopiridol and were maintained in culture without drug. Compared to parental cells, Flavo-R cells exposed to 0.2μM or 0.3μM flavopiridol continuously showed robust resistance to drug by a measure of the cell viability via annexin V-FITC and PI-PE by flow cytometry (Figure 1). These resistant cells develop the spontaneous resistance specifically to flavopiridol but not other therapeutics such as fludarabine (Supplementary Figure 1). Interestingly, Flavo-R developed the cross-resistance to dinaciclib which is a more potent and selective CDK inhibitor primarily targeting CDK1, 2, and 9 (IC50 of <5nM), implicating that these CDK inhibitors may target proximal pathways (Figure 1). [24, 25]

### Flavo-R exhibits high levels of transcriptional activity antagonizing flavopiridol's drug action

**Figure 2:**
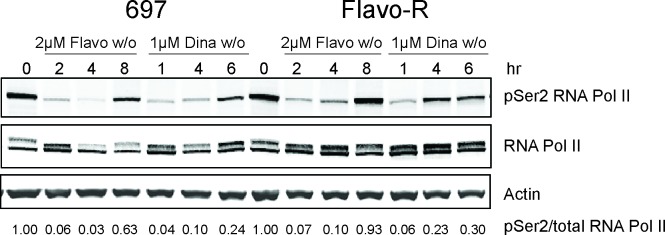
Phosphorylation of Ser2 of RNA Pol II CTD is more resistant against flavopiridol's drug action 697 parental and Flavo-R cells were treated with either 2μM flavopiridol with 4-hour exposure and washout (w/o), or 1μM dinaciclib with 2-hour exposure and washout (w/o) and collected at various time points as indicated in the figure. Protein lysates were prepared and subjected to immunoblotting for phosphorylation of Ser2 of RNA Pol II, total RNA Pol II and actin. Consistently, Flavo-R reveals more robust Ser2 phosphorylation with flavopiridol and dinaciclib, implicating higher activity of RNA Pol II. It also suggests that Flavo-R mechanistically establishes the resistance to dinaciclib in vitro in concert with observations in cell cytotoxicity described in Figure 1. Densitometry was applied to quantify the intensity of immunoreactive bands for phospho-Ser2 of RNA Pol II and, which was normalized to total RNA Pol II and the arbitrary numbers are shown at the bottom of the figure.

Flavopiridol has been shown to inhibit the phosphorylation of the C-terminal domain (CTD) of RNA polymerase II (RNA Pol II) and hence impede RNA transcription in cell-free system and in multiple cancers including CLL. [10, 26] RNA Pol II processes a large CTD, which consists of multiple repeats of heptamer sequence Tyr1–Ser2–Pro3–Thr4–Ser5–Pro6–Ser7. Concomitant with initiation and throughout the transcription cycle, the CTD becomes highly phosphorylated at Ser2 and Ser5 positions particularly. During transcription, CTD is initially phosphorylated on Ser5 by the CDK7/cyclin H and as RNA Pol II elongates, Ser2 is increasingly phosphorylated by CDK9/cyclin T, while Ser5 phosphorylation is gradually reduced by phosphatases. [27, 28] As flavopiridol was demonstrated to decrease the phosphorylation status of RNA Pol II CTD in primary CLL previously, phosphorylation of Ser2 and Ser5 sites was investigated in parental and Flavo-R cells. [10] Ser2 phosphorylation was greatly diminished upon flavopiridol treatment in parental cells while it appeared to be less affected in Flavo-R in a time course (Figure 2). After depletion of drug at 4-hour exposure of flavopiridol and 2-hour exposure of dinaciclib, Ser2 phosphorylation was quickly recovered in Flavo-R to promote RNA transcriptional machinery. Similarly, Flavo-R demonstrated sustained levels of Ser2 phosphorylation with dinaciclib, suggesting that CDK9 is the most relevant target of both therapies. Phosphorylation of the Ser5 site in Flavo-R was retained with flavopiridol, albeit to a lesser extent (Supplementary Figure 2). These data indicated that up-regulation of phosphorylation on both Ser2 and Ser5 of the CTD contributed to the observed resistant mechanism in Flavo-R and promoting cell survival against flavopiridol.

### Increased CDK9 activity with flavopiridol is associated with phosphorylation of Ser2 at RNA Pol II CTD in Flavo-R

**Figure 3:**
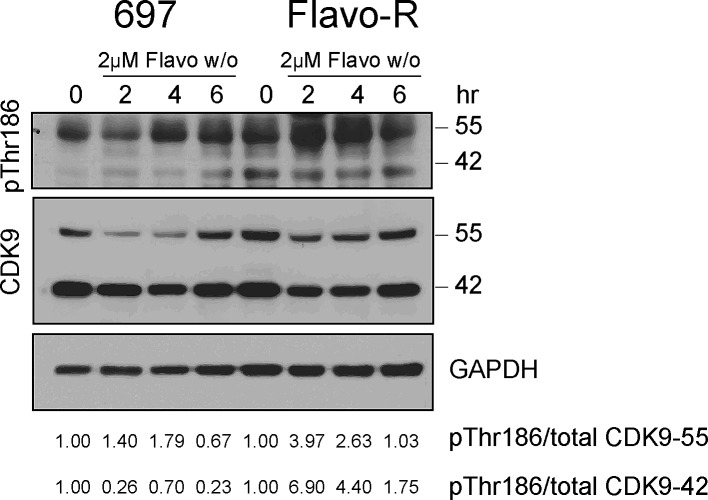
CDK9 kinase activity is upregulated to promote RNA Pol II activity, counter to the drug mechanism of flavopiridol 697 parental and resistant cells were treated with 4hr-exposure of 2μM flavopiridol and harvested for protein lysate at pre (0hr), 2, 4 and 6hr. Lysates were subjected to immunoblotting for phospho-Thr186 in the CDK9 activation loop as a surrogate for CDK9 kinase activity. Densitometry was applied to quantify the intensity of immunoreactive bands for phospho-Thr186 of both CDK9 isoforms, which were normalized to total CDK9 and the arbitrary numbers are shown at the bottom of the figure. CDK9 kinase activity of both isoforms was further increased with flavopiridol in Flavo-R.

As the results of enhanced phosphorylation of Ser2 in Flavo-R, we inspected whether the activity of the upstream kinase of Ser2 phosphorylation, CDK9, is upregulated with flavopiridol. CDK9/cyclin T promotes mRNA transcriptional elongation through phosphorylation of elongation repressors and RNA Pol II at Ser2. [25] As in other CDKs, phosphorylation has a major regulatory role in the CDK9 kinase activity and the conserved phosphorylation in the activation segment/T-loop at Thr186 is essential for CDK9 kinase activity. [29, 30] Moreover, CDK9 promoter has two start sites yielding two isoforms in mammalian cells, CDK9-42 and CDK9-55 where CDK9-55 has 117 additional amino acid residues in front of the amino terminus of CDK9-42. [31] These two CDK9 isoforms are differentially expressed and activated depending on the cell signaling stimuli, intracellular compartment and tissue types. [32-35] Therefore, we examined the CDK9 activity by detection of its activation loop phosphorylation at Thr186 of CDK9 in cells treated with 2μM flavopiridol and subjected to immunoblotting. Flavo-R exhibited prominent T-loop phosphorylation indicating up-regulation of CDK9 kinase activity in concert with the increased RNA transcription activity observed in these resistant cells (Figure 3). In addition, CDK9 protein levels were down-regulated with flavopiridol in both isoforms, suggesting that the drug mechanism of flavopiridol not only inhibits CDK9 kinase activity but also protein expression.

### CDK9 is an effective drug target of flavopiridol in vivo for CLL

**Figure 4:**
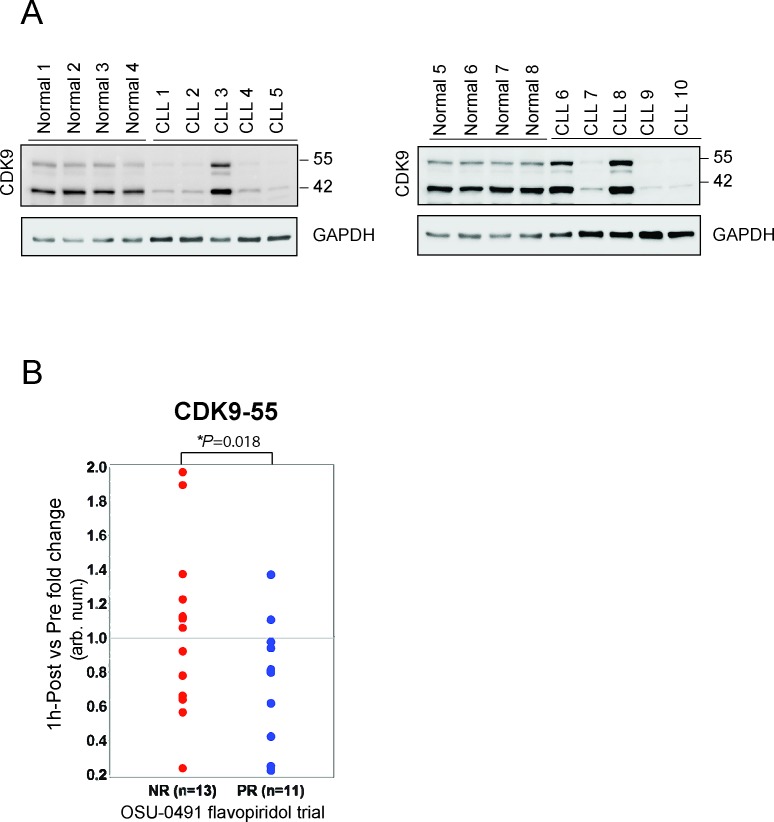
Reduction of CDK9-55 protein expression after flavopiridol therapy is correlated with the response in OSU-0491 clinical trial (A) Protein expression of both CDK9 isoforms is reduced in normal B-cells. CD19+ B-cells were selected from healthy donors or CLL patients and lysed for western blotting. Although the heterogeneity of protein expression is observed, overall CDK9 protein expression in CLL is less than in normal B-cells. (B) Protein lysates were prepared at pre and 1-hour post flavopiridol treatment collected on the OSU 0491 clinical trial. Comparison of CDK9-55 protein levels between post and pre showed significant decrease in partial responders but not in non-responders (*p*=0.018).

We further investigated the CDK9 protein expression in normal B-cell and B-CLL cells. CD19^+^ B-cells were isolated from leukopacks and CLL patients, lysed and immunoblotted for CDK9. CLL expressed significantly lower levels of CDK9 compared to normal donors (Figure 4A), suggesting a potential therapeutic index between these tumor cells and normal cells if targeting this is the predominate mechanism of action. We then compared in vivo CDK9 expression change between pre and post treatment of flavopiridol in samples collected from patients enrolled in the clinical trial, a phase II multicenter study (OSU-0491; NCT00098371). In this trial, flavopiridol was administered as a 30-minute loading dose followed by 4-hour continuous infusion in patients with previously treated CLL and B-CLL cells were isolated before and immediately after therapy at different time points. We examined the CDK9 expression in CLL cells before and one hour after infusion by immunoblotting and quantifying the immunoreactive band of CDK9 for densitometry. These data demonstrate that CDK9-55 expression between one-hour post and pre treatment is significantly downregulated in responders (n=11) as compared to non-responders (n=13; *p*=0.018) (Figure 4B). These data support that CDK9 could be one of the primary targets of flavopiridol in vivo that promote therapeutic efficacy of CLL.

### Flavopiridol-induced autophagy is elevated in Flavo-R but not ER stress response

Our previous data in CLL have shown that flavopiridol induces ER stress response pathways as one of its drug actions and activation of autophagy. [15] Concurrent treatment of flavopiridol and inhibition of autophagy by pharmacological agents or genetic knockdown of autophagy genes caused remarkable cytotoxicity in primary CLL. [15] We sought to examine if flavopiridol induces ER stress response or autophagy in the 697 cell line. Parental and Flavo-R cells were treated with vehicle, 2μM flavopiridol for 4-hour exposure, 1μM dinaciclib for 2-hour exposure, and 1μM thapsigargin, an authentic ER stress inducer as the positive control, collected for RNA isolation at various time points and measured by the real-time PCR with probes detecting ER stress response genes, including *IRE1*, *GRP78*, and *XBP1*. Thapsigargin treatment resulted in increased gene expression of *IRE1 and GRP78*, suggesting that 697 cell line is not compromised and susceptible to ER stress. However, unlike primary CLL, flavopiridol did not induce ER stress response in either 697 or Flavo-R cells (Supplementary Figure 3).

Next, flavopiridol-induced autophagy was examined by LC3B immunoblotting in parental and Flavo-R cells. Microtubule-associated protein 1 light chain 3B (LC3B) is a mammalian homolog of Atg8, which is involved in the autophagy biogenesis. During autophagy, a cytosolic form of LC3B (LC3B I) is cleaved and conjugated to phosphatidylethanolamine (PE) to form LC3B-PE conjugate (LC3B II), which is later recruited to autophagosomal membranes and subsequently degraded in the lysosome. Thus, the lysosomal turnover of LC3B II reflects induction of autophagy, and detection of LC3B by immunoblotting or immunofluorescence is used as a method for monitoring autophagy flux. [36, 37] However, increased LC3B II levels can be associated with either enhanced autophagy activity or reduced autophagosome turnover since autophagy is a dynamic process whereas autophagosme is constantly produced and processed. Hence, to better interpret changes in levels of LC3B II, cells were treated with flavopiridol and chloroquine, a lysosomal inhibitor that disrupts pH at the lysosome and impairs degradation of autolysosome, resulting in accumulation of LC3B II as an evidence of efficient autophagic flux. Paired cells were exposed to flavopiridol for 4 hours with or without 25μM chloroquine, collected for protein lysates and subjected to SDS-PAGE separation and immunoblotting for LC3B. LC3B II intensity was measured by densitometry, quantified, and normalized with GAPDH. Flavo-R displayed higher levels of autophagy mediated by flavopiridol than parental cells and the representative immunoblot is shown from 5 independent experiments (Supplementary Figure 4). However, reversal of this process in the cell line does not result in sensitization as seen with ER stress-mediated autophagy which was shown previously in primary CLL. [15]

### Increase of Mcl-1 stability with flavopiridol is observed in Flavo-R cells

**Figure 5:**
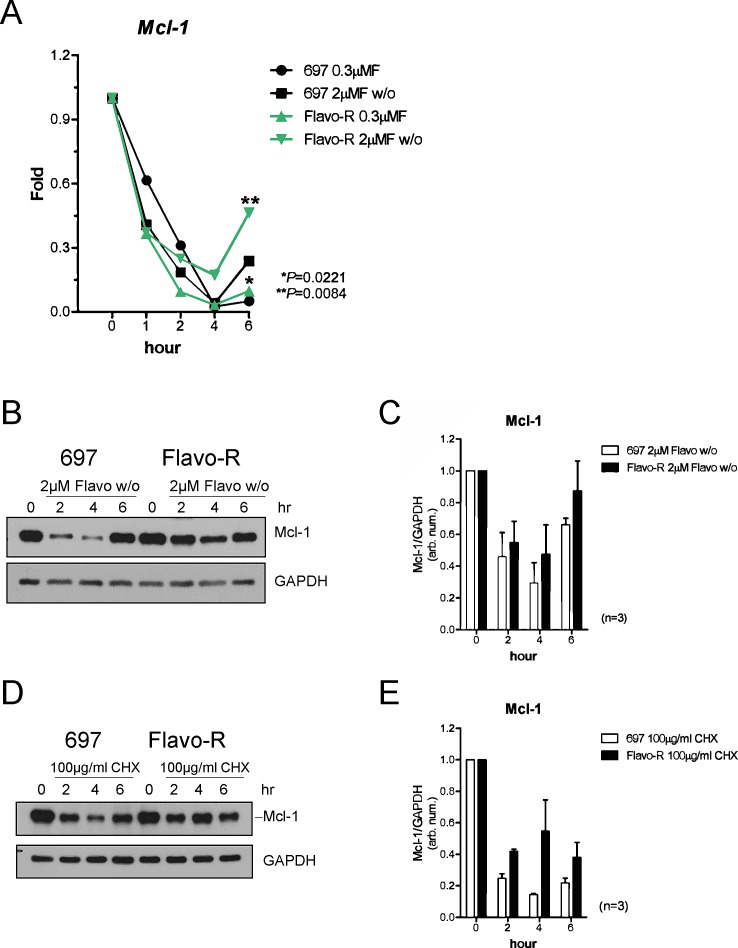
Mcl-1 protein levels are more stable to antagonize the flavopiridol-mediated depletion in Flavo-R (A) Cells were treated with continues exposure of 0.3μM flavopiridol or 2-hour exposure of 2μM flavopiridol with washout (w/o) and harvested for lysates and RNA preparation at pre (0 hr), 1, 2 and 4hr post treatment. Quantitative real-time PCR with TaqMan probes for *Mcl-1* was used to measure its transcript abundance after treatment. Flavo-R showed significantly more *Mcl-1* transcripts with both doses of flavopiridol at 6-hr time point. (B) Immunoblotting was applied to detect Mcl-1 protein levels in protein lysates collected from cells treated with 2μM flavopiridol for 4 hours and washout (w/o). (C) Densitometry is utilized to quantify the intensity of immunoreactive bands for Mcl-1 that is normalized to GAPDH and the bar graph shows the average of densitometry measurement of three independent experiments. Mcl-1 protein expression is more stable with the flavopiridol treatment in these resistant cells. (D) Cells were treated with 100μg/ml cycloheximide (CHX) to inhibit overall protein translation and compared with Mcl-1 protein stability between parental cells and Flavo-R. Treated cells were collected at pre (0h), 2, 4, and 6hr to assay short-life Mcl-1 protein levels by immunoblotting. (E) The bar graph shows the average of densitometry measurement of the intensity of immunoreactive bands for Mcl-1, which was normalized to GAPDH in three independent experiments. Levels of Mcl-1 protein expression are more stable in Flavo-R.

Flavopiridol-induced apoptosis has been associated with down-regulation of Mcl-1 in several cell systems. [10, 23, 38] Therefore, we investigated whether Mcl-1 contributed to the resistance to flavopiridol. Examination of Mcl-1 expression levels in parental and Flavo-R cells post drug treatment indicated increased Mcl-1 protein and transcript levels in Flavo-R cells (Figure 5). Parental and Flavo-R cells were treated with flavopiridol, harvested for RNA or protein isolation, and then analyzed by the real-time PCR or immunoblotting for Mcl-1. Significantly sustained levels of *Mcl-1* mRNA transcript were observed in Flavo-R cells following flavopiridol treatment at 6-hour time point (Figure 5A). In addition, Flavo-R cells exhibited more resistance to flavopiridol-mediated Mcl-1 protein degradation (Figure 5B and 5C). Stability of other anti-apoptotic proteins, Bcl-2 and Bcl-xL, were also examined; however, there is no difference with flavopiridol treatment between parental and Flavo-R cells. (Supplementary Figure 5) Our results showed that Flavo-R cells could overcome flavopiridol-induced cell death and developed resistance by elevating Mcl-1 levels. In all, our data implicate that these resistant cells are in part reminiscent of the clinical observations and can provide further understanding of resistant mechanism of flavopiridol.

### Up-regulation of Mcl-1 attributed to the translational stabilization through MAPK/ERK activation

Since Mcl-1 protein stability was increased and *Mcl-1* transcripts recovered quicker after flavopiridol removal in Flavo-R (Figure 5), therefore we examined the mechanism underlying the stability of Mcl-1 in these resistant cell lines. Mcl-1 has a very short half-life, estimated at less an hour, which can be seen with transcriptional inhibitor, actinomycin D (ActD). [39] Sensitive and resistant cell lines were treated with ActD, harvested, isolated for RNA and analyzed by the real-time PCR with probes for *Mcl-1*. We detected no differences in *Mcl-1* mRNA half-life, indicating that increased stability of Mcl-1 is not due to transcriptionally up-regulation of Mcl-1 levels in Flavo-R (Supplementary Figure 6). Based on these results, we examined whether upregulated Mcl-1 levels are due to the protein stabilization. Cells were treated with cycloheximide, a protein synthesis inhibitor used to measure the translational stability. Protein lysates were obtained after cycloheximide treatment from parental and resistant cells and performed immunoblotting for Mcl-1 (Figure 5D). We found that Mcl-1 protein levels are less susceptible to flavopiridol-mediated down-regulation in Flavo-R compared to the parental cell line. The average of densitometry measurement of Mcl-1 protein levels in three independent experiments is shown in Figure 5E.

**Figure 6:**
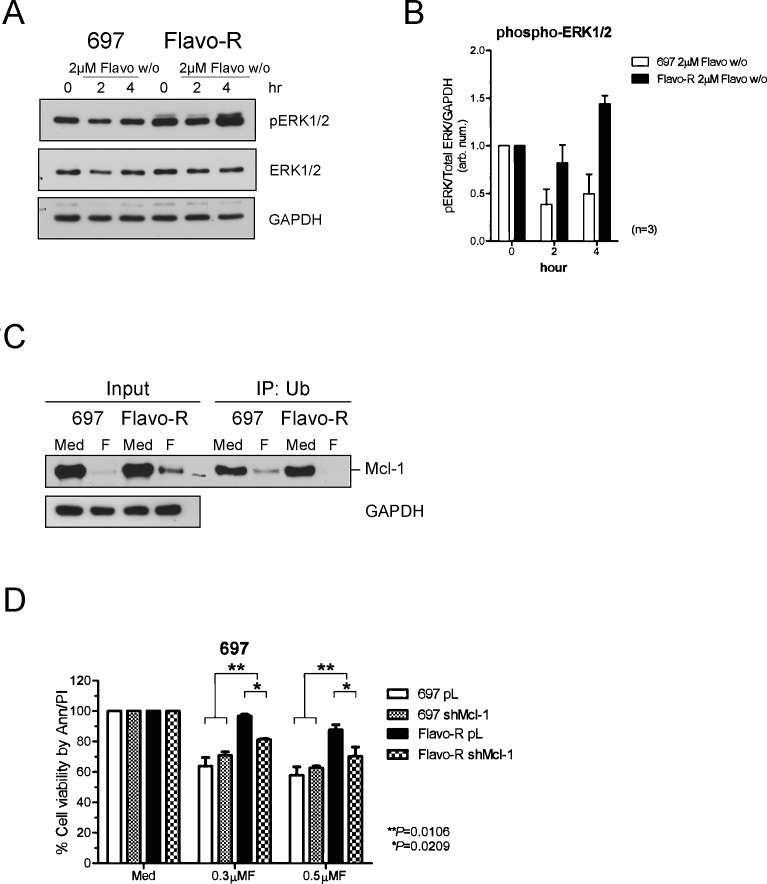
MAPK/ERK-mediated Mcl-1 stabilization contributes to resistance of flavopiridol and shRNA knockdown of Mcl-1 restores partial sensitivity to flavopiridol (A) Cells were treated with 4-hours 2μM flavopiridol with washout, harvested for protein lysates at pre (0h), 2, and 4hr, and yielded to immunoblotting for scrutinizing MAPK/ERK activation, which may contribute to preventing Mcl-1 from degradation. Phosphorylation of ERK was assayed by phospho-ERK1/2 antibodies to indicate the activation status of ERK kinase activity. (B) Densitometry was used to quantify the intensity of immunoreactive bands for phospho-ERK1/2, which was normalized to total ERK and GAPDH and the bar graph shows the average of densitometry measurement of three independent experiments. (C) Ubiquitinated proteins were pulled down with the ubiquitin-affinity agarose, subjected to SDS-PAGE separation and immunoblotted with the Mcl-1 antibody. The level of ubiquitinated Mcl-1 is reduced in Flavo-R with 4-hour exposure of 2μM flavopiridol, implicating that Mcl-1 in Flavo-R is less subjected to ubiquitin-conjugated degradation. (D) Stable clones for *Mcl-1* and control knockdown in 697 and Flavo-R cells were treated with 0.3μM or 0.5μM flavopiridol continuously and assayed for the cellular apoptosis 24-hours post treatment with annexin V-FITC and PI-PE stains analyzed by flow cytometry.

Inasmuch as increased Mcl-1 levels in Flavo-R are attributed to the translational stabilization, we further investigated if this stabilization results from activation of the mitogen-activated protein kinase (MAPK/ERK)-modulated pathway. Phosphorylation of Thr163 of Mcl-1 in the PEST region by ERK has been shown to slow turnover of Mcl-1 protein. [40] PEST sequence is enriched in proline, glutamic acid, serine, and threonine and is associated with proteins subjected to rapid degradation via the proteasome pathway. Moreover, Pepper *et al.* have demonstrated that flavopiridol decreases phosphorylation of ERK in CLL 24hr post-in vitro treatment measured by flow cytometry. [41] Thus, we scrutinize whether ERK activity is upregulated to stabilize Mcl-1 in Flavo-R. The activity of ERK was assessed by its activation loop phosphorylation at Thr202/Tyr204 and Thr185/Tyr187 of ERK1 and ERK2 respectively (Figure 6A and 6B). Increase of ERK phosphorylation with flavopiridol is correlated with more stabilized Mcl-1 protein levels in Flavo-R. Therefore, we further examine if ERK phosphorylates Mcl-1 to promote its protein stabilization and prevent it from the ubiquitin-conjugated degradation pathway. Due to a lack of commercial available antibodies to recognize the ERK-mediated phosphorylation at Thr-163 on PEST domain of Mcl-1, we alternatively exploited the immunoprecipitation-western blotting to measure the levels of ubiquitinated Mcl-1 in parental and Flavo-R cells. Ubiquitinated proteins were pulled down with the ubiquitin-affinity agarose from total lysates isolated from pre and 4-hour exposure of 2μM flavopiridol, yielded to SDS-PAGE separation and immunoblotting for Mcl-1. Lower levels of ubiquitinated-Mcl-1 were observed in Flavo-R compared to parental cells, implicating that Mcl-1 in Flavo-R is less subjected to ubiquitin-conjugated degradation. (Figure 6C)

### Mcl-1 knockdown restores partial sensitivity to flavopiridol

The translational stabilization of Mcl-1 is shown to be associated with the resistant to flavopiridol in Flavo-R. Thus, we investigated whether reducing levels of Mcl-1 could sensitize Flavo-R again via the therapeutic agent or genetic knockdown. MEK inhibitor, CI-1040, was used to inhibit the mitogen-activated protein kinase kinases (MEK) and the downstream MAPK/ERK activities to decrease phosphorylation of Mcl-1 and hence its stability. However, combination of CI-1040, and flavopiridol had no effect on re-sensitizing Flavo-R via the measure of the cell viability due to the ineffective inhibition of ERK1/2 activity by CI-1040 (data not shown). We then used alternative approach to infect parental and Flavo-R cells with specific shRNA constructs targeting *Mcl-1* as well as a control. After puromycin selection, we were able to identify a stable clone, sh*Mcl-1* 390 that exhibited a significant knockdown of Mcl-1 compared to the control for further analyses (Supplementary Figure 7). We then examined the sensitivity of Flavo-R with sh*Mcl-1* knockdown to flavopiridol compared to the control. Although, sh*Mcl-1* knockdown did not completely restore the sensitivity to flavopiridol to parental levels, reduction of Mcl-1 levels did result in more cell death in 697 flavopiridol-resistant cells (*p*=0.0106) (Figure 6D). These results suggest that while Mcl-1 up-regulation is important to acquire resistance in Flavo-R, other mechanisms or targets, like CDK9-associated pathways may also participate.

## DISCUSSION

Flavopiridol has shown promise particularly in CLL with potent cytotoxicity and effectiveness with genetically high-risk patients, especially those with del(17p13.1) and del(11q22.3). [9, 11, 12] Thus, flavopiridol and other cyclin-dependent kinase inhibitors are pursued as single agents for the frontline treatment for patients with poor-risk cytogenetic abnormalities, as well as combination therapies in relapsed/refractory disease. Despite few clinical reports on the correlation of response and clinical trials, it is largely unknown about the resistance mechanism of flavopiridol. The acquired resistance is poorly understood and is currently approached by correlating the inherent genetics with responses to stratify patients for clinical purposes. However, the mechanism of acquired resistance to therapies may be different from the cause before treatment and specifically developed in response to this treatment. Therefore, the study herein is an attempt to understand the molecular basis of acquired resistance to flavopiridol in a leukemia model to provide insight in clinically relevant understanding for refractory/relapsed patients.

In our flavopiridol-resistant leukemia cell line model, our results implicate that selection for elevated CDK9 kinase activity, upregulated transcriptional activity, induction of autophagy, and increase of Mcl-l stability concomitantly contributes to resistance to flavopiridol. CDK9 has been reported as the primary target of flavopiridol as well as down-regulation of RNA transcription activity and Mcl-1 levels. [10, 15, 17, 23] In concert with these findings, the resistant cells demonstrate enhanced CDK9 activity and hence RNA Pol II activity antagonistic toward flavopiridol's actions, emphasizing the importance of these targeted pathways in flavopiridol-mediated cell death. Our data also suggest that flavopiridol not only inhibits phosphorylation of RNA Pol II but also its upstream kinase CDK9 in terms of its protein expression and kinase activity to potentiate its capability in induction of cytotoxicity. Notably, change of CDK9-55 protein expression after therapy inversely correlates with response to flavopiridol, indicating CDK9 is the primary therapeutic target in vivo. It has been demonstrated that CDK9-55 expression is associated with the regulation of the cell differentiation program of various tissues, such as the hematopoietic compartment and muscles. [33, 42, 43] However, limited data are available regarding the function of CDK9-55 separately from CDK9-42 in hematopoietic and lymphoid tissues. Further study is necessary to delineate the role of CDK9-55 and its related pathways in CLL to fully characterize the molecular actions of flavopiridol and to improve CDK9-targeted therapies. In addition to the phosphorylation of the RNA Pol II CTD, CDK9 has been shown to be involved in the transcription regulation of NFκB and STAT3 and interaction with a variety of factors, such as retinoblastoma (Rb), p53 and c-Myc that may also contribute to the resistance of flavopiridol and are worthy of investigation to delineate the molecular mechanism of the drug action. [44-47]

Furthermore, increase of Mcl-1 protein stability is observed in this leukemia model, suggesting the Mcl-1 protein level directly contributes to the resistance of flavopiridol. Mcl-1 is an essential survival factor in lymphocytes supported by a mouse model conditional for *Mcl-1* that displays a profound reduction in B and T lymphocytes. [48] An inverse correlation was found between Mcl-1 expression and Rai stage and sensitivities of the leukemic cells to chemotherapies in CLL. [49] Our data show that resistant cells maintained substantial levels of Mcl-1 protein with flavopiridol, which is associated with ERK activation. Cycloheximide treatment determined that Mcl-1 is translationally more stable in the resistant cells in accordance with the elevated ERK activity and the reduced ubiquitin modification detected in Flavo-R. In concert with previous findings, our data demonstrate that increased translational stabilization of Mcl-1 is mediated by ERK phosphorylation to prevent ubiquitin-dependent degradation. Furthermore, knockdown of *Mcl-1* partially restores the sensitivity in Flavo-R, validating that Mcl-1 is a key controller of drug resistance.

Interestingly, Flavo-R shows cross-resistance to another CDK inhibitor, dinaciclib while exposed to 1μM concentration for 2 hours and washed off. Dinaciclib is a more selective CDK inhibitor of CDK1, CDK2, CDK5, and CDK9 with IC50 of <5nM in an in vitro kinase assay and currently under development of phase III clinical trial in CLL. Compared with flavopiridol in an in vitro kinase assay, dinaciclib inhibits CDK1 and CDK9 equally but CDK2 and CDK5 with more than 10-fold potency. [24] Likewise, dinaciclib demonstrated remarkable cytotoxicity in CLL and in vivo inhibition of Mcl-1 expression and PARP cleavage in acute myeloid leukemia patients. Preclinical data in melanoma mouse xenografts and osteosarcoma cells, showed that dinaciclib induces apoptosis, increases Bax and Bim in mitochondria, and decease antiapoptotic factors, such as Mcl-1, Bcl-2, Bcl-xL and XIAP, as well as phosphorylation of Ser2 of RNA Pol II. [25, 50-52] This is also supported by our findings that the flavopiridol-resistant cells became insusceptible to dinaciclib and displayed increased levels of Mcl-1 and P-TEFb activity while treated with this drug. Thus, these results suggest that both flavopiridol and dinaciclib initiate the apoptotic effects primarily through inactivation of CDK9 and transcription in concert with down-regulation of Mcl-1.

Taken together, we demonstrate the mechanisms of resistance that are selected specifically for the acquired resistance to flavopiridol in the leukemia cells. The investigation of resistance mechanisms reveals that increased levels of Mcl-1 and CDK9 activity attribute to overcoming flavopiridol-induced cell death. Combined with the results from clinical trials with flavopiridol, it is apparently favorable to continuously develop therapies targeting CDK9 in CLL. In all, our collective data suggest that future studies are warranted to scrutinize targeted therapies for Mcl-1 and CDK9-associated pathways to combat the resistance to flavopiridol.

## MATERIALS AND METHODS

### Generation of flavopiridol-resistant (Flavo-R) cell lines

The 697 cell line was obtained from American Type Culture Collection (ATCC) and cultured in RPMI-1640 medium (Life Technologies, Grand Island, NY) supplemented with 10% FBS, _L_-glutamine and antibiotics (Life Technologies). 697 cells were treated with gradually increasing doses of flavopiridol, with the drug being eluted off to allow recovery. 697 Flavo-R was eventually cultured to be resistant to flavopiridol at 0.5μM. Cells were examined for the intracellular drug concentration of flavopiridol to exclude the possibility of acquiring resistance due to up-regulation of drug efflux or accumulation (data not shown). [53] Cytogenetic characterization of parental and resistant cells revealed similar patterns (Supplemental Table 1).

### Patients, cell separation, chemical reagents, and cell treatment

For both in vivo and in vitro studies, written, informed consent was obtained in accordance with the Declaration of Helsinki to procure cells from patients diagnosed with CLL as defined by the modified iwCLL 2008 criteria. [54] Approval was obtained from The Ohio State University (OSU) institutional review board. CD19^+^ cells from CLL patients, and healthy volunteers were isolated and maintained in culture as previously described. [15] Flavopiridol (alvocidib) and dinaciclib (SCH 727965) were obtained from the National Cancer Institute. Both were dissolved in dimethylsulfoxide at 22.8mM and 10mM respectively, and stored at −80°C in small aliquots. Cells were treated with flavopiridol in RPMI-1640 medium supplemented with 10% human serum (Valley Biomedical, Winchester, VA), and _L_-glutamine and antibiotics. In order to resemble the clinical administration schedule for correlating pharmacodynamics markers and findings from our in vitro studies to clinical data, we expose cells 2μM flavopiridol for 4 hours or 1μM dinaciclib for 2-hour exposure with the wash out procedure if the time point is longer than 4 or 2 hours respectively. [7, 12, 25] Thapsigargin, chloroquine, actinomycin D, cycloheximide, fludarabine, CI-1040, and puromycin were purchased from Sigma-Aldrich, St. Louis, MO.

### Immunoblotting and immunoprecipitation (IP)-Western

As described previously, cells were lysed, sonicated and protein concentration was quantified by BCA analysis (Thermo Scientific, Rockford, IL). 50μg of protein lysate was resolved on SDS-PAGE, transferred to PVDF membrane, and blotted with specific primary and secondary antibodies (Sigma-Aldrich) accordingly. [15] For IP-Western, 500μg of protein lysate was incubated with Ubiquilin 1 tandem UBA (TUBE2) agarose (Boston Biochem, Cambridge, MA) in the IP buffer (25mM Tris•HCl, pH7.4, 150mM NaCl and 1% NP-40) on a rotation platform at 4ºC overnight. Ubiquitinated proteins were bound to the agarose, washed three times with IP buffer and subjected to immunoblotting. [55, 56] Primary antibodies were used for Mcl-1, CDK9, actin (Santa Cruz Biotechnology, Dallas, TX), phospho-Pol II CTD at Ser2 and Ser5, Pol II CTD (Covance, Princeton, NJ), phospho-ERK1/2, ERK1/2, phospho-CDK9, phospho-Bcl-2, Bcl-xL (Cell Signaling Technology, Danvers, MA), Bcl-2 (Dako, Carpinteria, CA) and GAPDH (EMD Millipore, Billerica, MA). Densitometry of immunoreactive bands on immunoblots was measured with FluorChem E System (ProteinSimple, Santa Clara, CA), quantified and normalized with AlphaView (ProteinSimple).

### RNA isolation and quantitative real-time PCR

Cells were homogenized with TRIzol reagent (Life Technologies) and isolated for RNA by RNeasy Mini Kit (Qiagen, Valencia, CA). cDNA was prepared with random hexamers (Life Technologies) and M-MLV reverse transcriptase (Life Technologies). Gene expression was measured by the quantitative real-time PCR with TaqMan gene expression assays for *Mcl-1 and CD52* (Life Technologies) and detected by ViiA 7 Real-Time PCR System (Life Technologies). Average relative expression (treatment compared to vehicle) was normalized to the endogenous control gene, *CD52*, and calculated by the comparative Ct (cycle number required to reach detection threshold) method, known as 2^−(ΔΔCt)^ method. [57] *Mcl-1* mRNA stability was determined following 2μg/ml actinomycin D treatment and the real-time PCR analysis.

### Cell apoptosis assays

Cell death was measured 24-hour post treatment with fluorescein-conjugated annexin V and propidium iodide (PI) (BD Biosciences, San Jose, CA) and analyzed by a FC500 flow cytometer (Beckman Coulter, Indianapolis, IN) as described previously. [24, 25] Results were normalized to control cells that had been treated with vehicle.

### Generation of lentiviral-based knockdown cell lines

*Mcl-1* knockdown was achieved in 697 cells with short hairpin RNAs (shRNAs) by lentiviral transduction using calcium phosphate method (Promega, Madison, WI) according to Byrd lab protocol modified from *Current Protocol in Molecular Biology*. [58] Knockdown clones with stable expression of pLKO.1 vector (Thermo Scientific) or *Mcl-1* shRNAs (Sigma-Aldrich) were selected and cultured with puromycin.

### Statistical analysis

Mixed effects models were used for experiments involving cell viability (Flavo-R versus parental cells as well as in the presence of sh*Mcl-1* knockdown), *Mcl-1* expression in Flavo-R and parental cell line, and CDK9-55 protein expression. For the cell viability experiments, viability (% of annexin V and PI negative cells relative to media) in the presence of either flavopiridol or dinaciclib was compared between 697 Flavo-R and 697 separately. Due to the similar effects of continuous 0.2μM, 0.3μM flavopiridol, *p*-values for the average effect over both doses are presented, rather than for each dose separately. Similarly, the effect of sh*Mcl-1* knockdown on cell viability in the presence of flavopiridol was calculated from the mixed effects model; the average effect of 0.3μM and 0.5μM flavopiridol was estimated for both parental and Flavo-R cell lines separately, and an interaction contrast was used to assess the relative effect between the two cell lines. For *Mcl-1* expression, differences in fold change at 6-hour post-treatment between the Flavo-R and parental cells were estimated. CDK9-55 expression between one-hour post and pre-treatment was compared between partial responders and non-responders using an interaction contrast from the mixed effects model; data were first natural log-transformed to stabilize variances. All analyses were performed using SAS/STAT software, version 9.2 (SAS Institute Inc., Cary, NC).

## SUPPLEMENTARY MATERIAL, TABLE AND FIGURES


