# Supplementary material for: Up-regulation of CDK9 kinase activity and Mcl-1 stability contributes to the acquired resistance to cyclin-dependent kinase inhibitors in leukemia
Source: Oncotarget. 2014 Jun 12;6(5):2667–79. doi: 10.18632/oncotarget.2096 (PMC4413609; doi:10.18632/oncotarget.2096)
Supplement: Supplementary file 1 [file oncotarget-06-2667-s001.pdf]

Up-regulation of CDK9 kinase activity and Mcl-1 stability contributes to the acquired resistance to cyclin-dependent kinase inhibitors in leukemia

Supplementary Materials

Table S1: Cytogenetic test of 697 parental and resistant cell lines by fluorescence *in situ* hybridization (FISH).

**ISCN of cell lines**	
**697 parental**	46,XY,der(4)t(4;8)(p15.2;q11.2),inv(5)(q13q35),del(6)(q15q23),del(10)(q25.2q26.1),der(19)t(1;19)(q23;p13.3)[2]/46,sl,inv(16)(p13.3q11.2)[18]
**697 Flavo-R**	46,XY,der(4)t(4;8)(p15.2;q11.2),inv(5)(q13q35),del(6)(q15q23),del(10)(q25.2q26.1),der(19)t(1;19)(q23;p13.3)[20]

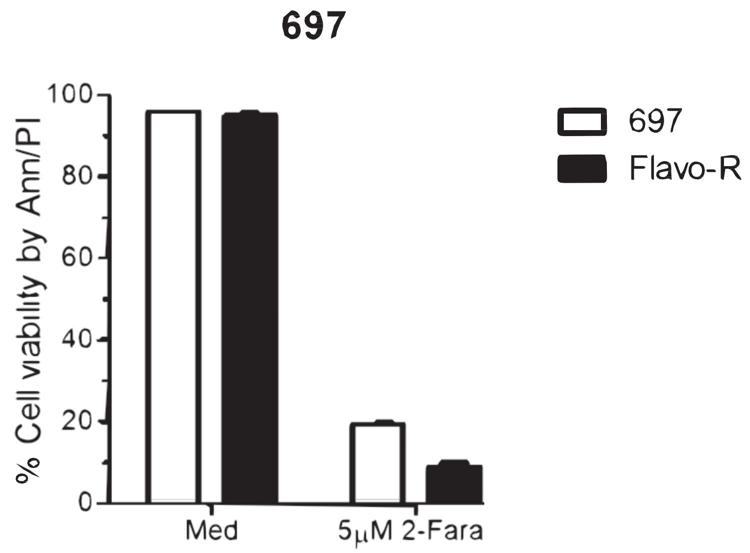

Figure S1: Fludarabine induces potent cytotoxicity in 697 and Flavo-R cells. Cells were treated with vehicle or 5μM fludarabine (2-Fara) for 24 hours and cell viability was measured by annexin V-FITC and PI-PE stains with Tali Image Cytometer (Life Technologies).

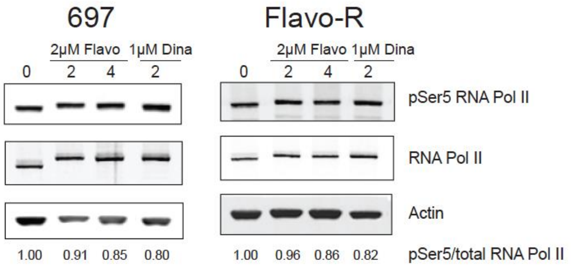

Figure S2: Phosphorylation of Ser5 of RNA Pol II CTD is relatively stable with the flavopiridol treatment. 697 parental and Flavo-R cells were treated with either 2μM flavopiridol or 1μM dinaciclib and collected at various time points as indicated in the figure. Protein lysates were prepared and subjected to immunoblotting for phosphorylation of Ser5 of RNA Pol II, total RNA Pol II and actin. Flavo-R shows relatively more Ser5 phosphorylation with flavopiridol and dinaciclib, however in lesser extent. Densitometry was applied to quantify the intensity of immunoreactive bands for phosphor-Ser5 of RNA Pol II and, which was normalized to total RNA Pol II and arbitrary numbers are shown at the bottom of the figure.

A

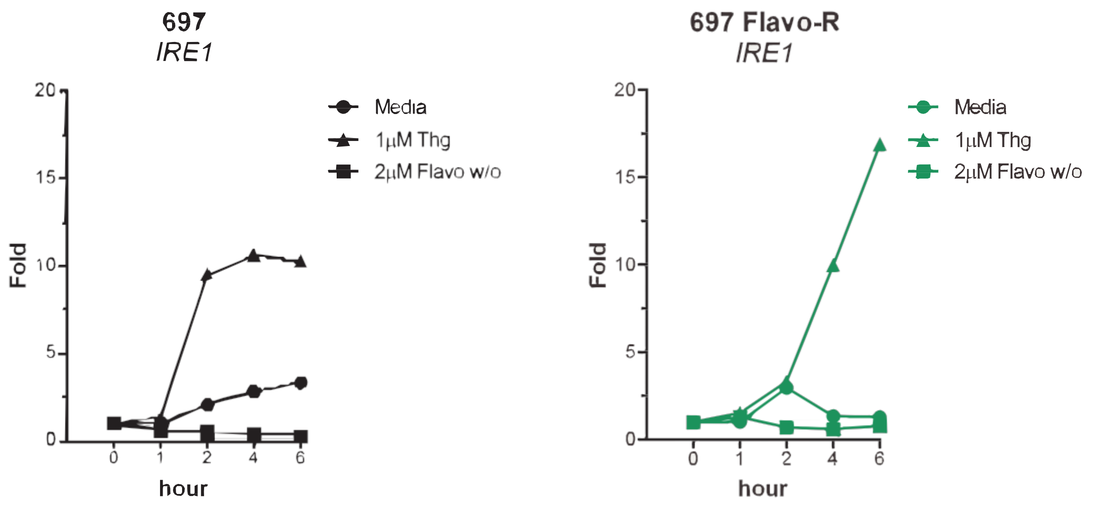

B

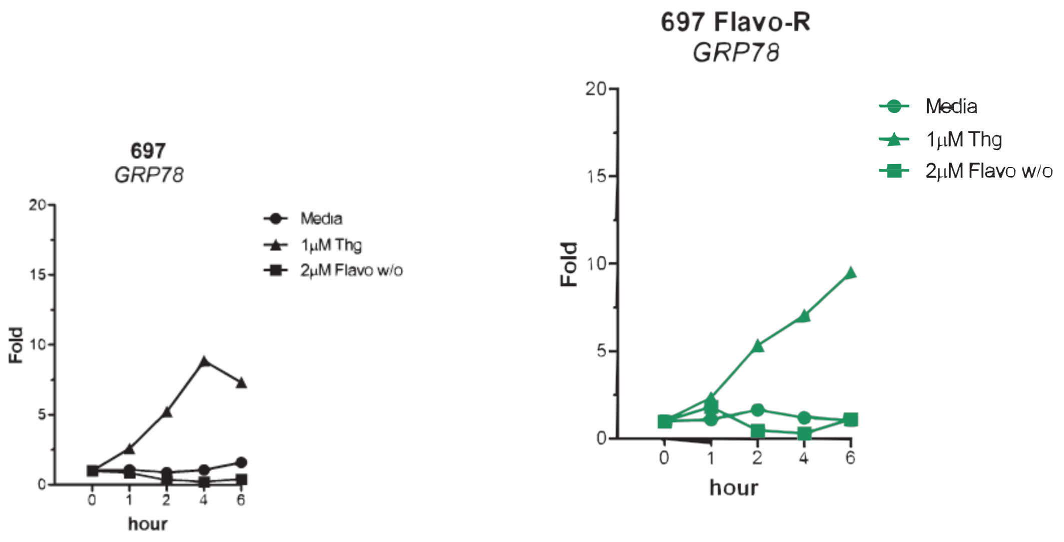

Figure S3: Flavopiridol does not induce ER stress response in 697 cell line. Cells were treated with vehicle, 2μM flavopiridol for 4 hours and washout, and 1μM thapsigargin and collected at various time points as indicated to isolate RNA for the real-time PCR analysis detecting *IRE1* and *GRP78* transcript levels. Thapsigargin is the classic ER stress inducer which induced increase of ER stress response genes in both cell lines; however, unlike in primary CLL, flavopiridol treatment did not result in induction of ER stress response.

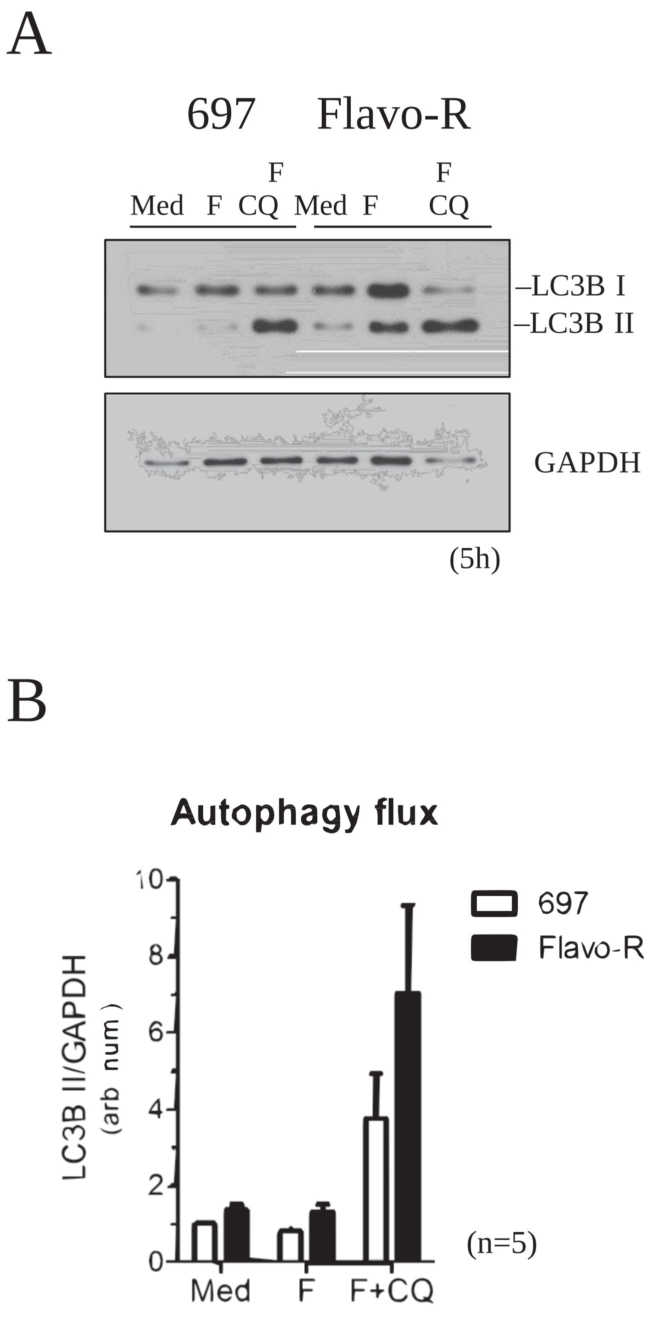

Figure S4: Elevated levels of autophagy activity are induced by flavopiridol. (A) Cells were exposed to 2μM flavopiridol for 4 hours and washout with or without chloroquine, collected for lysates at 5 hours and subjected to immunoblotting for LC3B II, a measure of autophagy induction. (B) Independent experiments were performed for 5 times and quantified by densitometry as shown in the figure. More LC3B II accumulation was observed in Flavo-R, suggesting the increase of the autophagy flux.

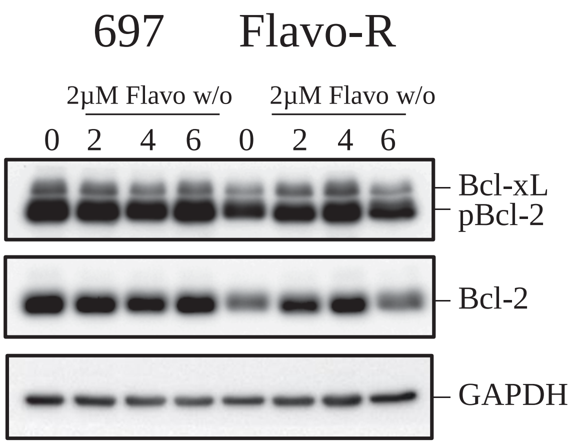

Figure S5: Levels of anti-apoptotic proteins, Bcl-2 and Bcl-xL are comparable in both cell lines. Cells were treated with 2μM flavopiridol for 4 hours and washout and collected for protein lysates. Protein expression of phospho-Bcl-2, Bcl-2 and Bcl-xL was detected by immunoblotting and exhibited no difference between parental and Flavo-R cells.

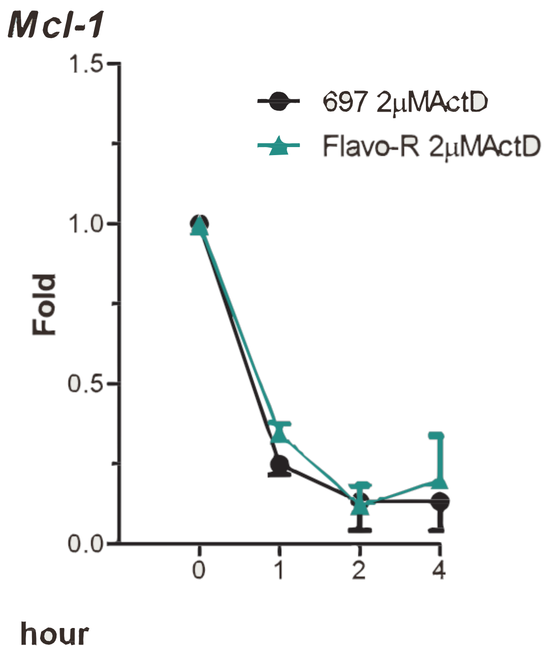

Figure S6: Stability of Mcl-1 observed in Flavo-R is enhanced through translational stabilization but not transcriptional control. Cells were incubated with 2µM actinomycin D (ActD) to inhibit global RNA transcription and isolated for RNA at various time points to compare *Mcl-1* transcript levels between parental and Flavo-R via the real-time PCR. There was no difference detected in *Mcl-1* decay between parental and Flavo-R cells. Cells were 80 – 100% viable at 4-hour post treatment analyzed by annexin V-FITC and PI-PE stains with Tali Image Cytometer.

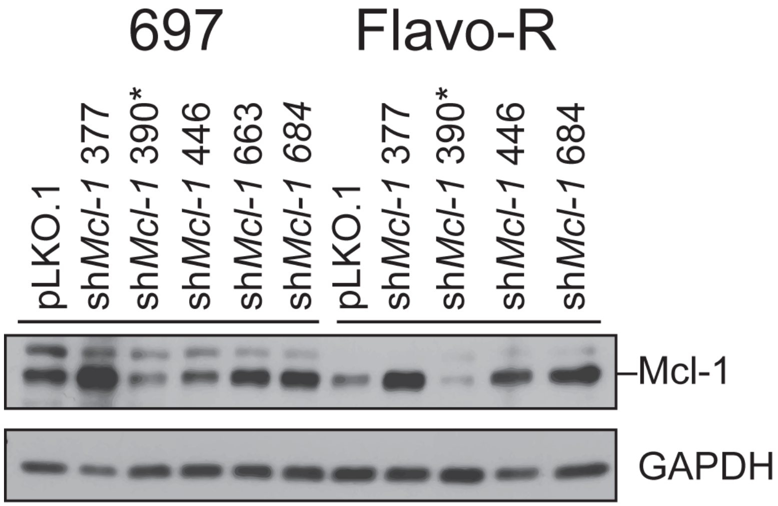

Figure S7: Stable clones for *Mcl-1* shRNA knockdown in parental and resistance cell lines show relatively minimum Mcl-1 protein expression to the control.

Stable knockdown of *Mcl-1* clones was achieved by lentiviral transduction and followed by puromycin selection for a period of time. Protein lysates were prepared from each knockdown clone and yielded for immunoblotting for Mcl-1. sh*Mcl-1* 390 clone (*asterisk) was selected and used for the further analysis described in this study.
